# The tapeworm species recorded in carnivores of Slovakian wildlife - molecular analyses

**DOI:** 10.3389/fvets.2026.1804086

**Published:** 2026-05-08

**Authors:** Jozef Lazár, Júlia Šmigová, L'ubomír Šmiga, Viliam Šnábel, Yaroslav Syrota, Ladislav Molnár, Marián Chabada, Mikuláš Hančin, Ingrid Papajová, Peter Lazár

**Affiliations:** 1 Department of Breeding and Diseases of Game, Fish and Bees, Ecology and Cynology, University of Veterinary Medicine and Pharmacy in Košice, Košice, Slovak Republic; 2 Slovak Academy of Sciences, Institute of Parasitology, Košice, Slovak Republic; 3 I. I. Schmalhausen Institute of Zoology NAS of Ukraine, Kyiv, Ukraine; 4 University Veterinary Hospital/Clinic of Birds, Exotic and Wild Living Animals, University of Veterinary Medicine and Pharmacy in Košice, Košice, Slovak Republic; 5 State Nature Conservation of the Slovak Republic, Banská Bystrica, Slovak Republic

**Keywords:** canids, felids, Slovak Republic, Taeniidae, wildlife

## Abstract

**Introduction:**

Important cestode species parasitizing carnivores are the worldwide distributed tapeworms of the family Taeniidae. Typical for taeniids is an indirect life cycle involving two obligate mammalian hosts in a predator-prey scheme, with an herbivore intermediate host and a terrestrial carnivore definitive host. The aim of the study was the molecular characterization of taeniid tapeworms from wild and domestic carnivores obtained from a national park and hunting areas from three different districts of eastern Slovakia.

**Methods:**

Five tapeworm samples from canids and felids definitive were examined by sequences of regions of 463 bp and 396 bp in the *cox1* gene, which were subjected to phylogenetic analysis.

**Results:**

Two *Taenia* species were genetically confirmed from carnivores in Slovakia. The record of *Taenia lynciscapreoli* found in the Eurasian lynx (*Lynx lynx*) in the Tatra National Park represents the first finding of this species in Slovakia. The four isolates obtained from canids - from the gray wolf (*Canis lupus*), golden jackal (*Canis aureus*), and domestic dog (*Canis lupus familiaris*) belonged to *Taenia krabbei*. These isolates were distributed at three different positions in the cluster attributed to *T. krabbei*, partitioned according to their different definitive hosts (wolf, dog, jackal).

**Conclusion:**

Although the original and dominant definitive host of *T. krabbei* is the wolf, half of the *T. krabbei* samples were collected from non-wolf definitive hosts. The finding of *T. krabbei* in a single isolate from a free-ranging dog in a hunting area corroborated the potential role of this host in the life cycle of this species.

## Introduction

Tapeworms of the genus *Taenia* Linnaeus, 1758 (Cestoda: Taeniidae) are intestinal parasites commonly found in wild carnivores such as wolves (*Canis lupus* Linnaeus, 1758), jackals (*Canis aureus* Linnaeus, 1758), or lynxes (*Lynx lynx* Linnaeus, 1758). Taeniids are characterized by an indirect life cycle including two obligate mammalian hosts: the definitive host, infected through predator-prey relationships (1, 2), harbors the adult parasite in the small intestine, while the larval stages (metacestodes) develop in various tissues (e.g., muscle, liver) and/or body cavities (omentum, mesentery, peritoneum) of their intermediate hosts, such as wild ruminants (family Cervidae) or wild boars (3).

Tapeworms from the family Taeniidae, with a global distribution, represent an important species of cestodes of canines and felids. These intestinal parasites play a significant role in wildlife parasitology, but they also pose a zoonotic concern due to affecting the meat of intermediate hosts and potential spillover of infection to domestic canids or felids living in close proximity to humans (4). Adult tapeworms are generally considered to be of low pathogenicity, and infected intermediate hosts usually remain asymptomatic and do not show overt clinical signs of disease (5). Therefore, this parasitic infection is usually only revealed after the post-mortem examination.

The typical definitive hosts of the species *Taenia krabbei* (44) are members of the family Canidae, especially wolf (*Canis lupus*) (6). The main intermediate hosts are represented by wild cervids. In Europe, the occurrence of *T. krabbei* in wild carnivores has been confirmed in Germany (6), Italy (7), Sweden and Norway (8), and the Czech Republic (9). In Slovakia, to our knowledge, *T. krabbei* was recorded in wolves, red foxes and red deer (*Cervus elaphus* Linnaeus, 1758) in a single study assessing the prevalence of adult and larval stages of *Taenia* in 437 wild animals, conducted in the first decade of this century (10).

The typical definitive host of the species *Teania lynciscapreoli* (11) is the Eurasian lynx (*Lynx lynx*). The main intermediate host is roe deer (*Capreolus capreolus* Linnaeus, 1758), but metacestodes have also been found in Siberian roe deer in Russia (*Capreolus pygargus* Pallas, 1771), Eurasian moose (*Alces alces* Linnaeus, 1758) in Finland (11), and in semi-domesticated reindeer from Sweden (*Rangifer tarandus tarandus* Linnaeus, 1758) (4). The metacestodes of *T. lynciscapreoli* encyst in the lungs of intermediate host with no other site of infection reported to date (4, 11). The spread of the parasite indicates a southeastern Eurasian distribution, which appears to be most dependent on hunting roe deer, the preferred prey of the lynx (12). *Taenia lynciscapreoli* has not been found in lynx outside the range of the roe deer, suggesting a transmission route based on a specific predator-prey relationship (11).

No previous molecular studies have been conducted on this topic in Slovakia, perhaps also due to the fact that obtaining samples from protected wild animals, such as lynxes, is quite difficult. Parasite specimens derived from these animals are scarce and any information derived from these samples is valuable. Consequently, the primary objective of this study was the molecular characterization of taeniids obtained from the carcasses of wild carnivores, collected with the cooperation of the Nature Conservation authorities. The study was undertaken to better understand the distribution of *Taenia* spp. in these hosts in Slovakia and to indicate the likelihood of multi-host infections in areas where the ranges of wild and domestic definitive hosts overlap.

## Material and methods

### Samples and sampling

We performed a post-mortem examination on four specimens of the family Canidae - two adult males from the gray wolves, one adult female from the golden jackal, one adult male from the feral street dog (*Canis lupus familiaris*, Linnaeus, 1758), and of the Felidae family - one adult female from the Eurasian lynx. The carnivores came from eastern Slovakia, and they were collected by the employees of the State Nature Conservation of the Slovak Republic. Wolf samples were taken from dead animals; the carcasses of adult males were found in the district of Bardejov (near the village Beloveža) and in the district of Poprad (near the village Nová Lesná), both located in the northeastern part of the country. Other canid samples came from the Košice district; an adult female of jackal was killed near the village of Rozhanovce in 2023, and an adult male domestic dog in a hunting ground located near the village of Mudrovce in 2024. The carcass of an adult lynx female was found in the Tatra National Park (Poprad – Starý Smokovec). All observed animals were in poor body condition, with the exception of the jackal. The taeniid cestodes were found in the intestines during the procedure of evisceration. This process was performed by the authors themselves in the necropsy room of the University Veterinary Hospital, Clinic of Birds, Exotic and Wild Living Animals of the University of Veterinary Medicine and Pharmacy in Košice. The collected tapeworms were washed with physiological solution and then stored in 96% alcohol for molecular analyses.

The stored tapeworms were counted, washed with physiological solution, and the worm bodies were cleared with lactophenol to examine their morphological structures according to morphological keys in the Global Cestode Database (13). The lynx isolate was consistent with *T. lynciscapreoli* because it had shorter hooks than other congeneric species parasitizing felids in the Holarctic region (11, 13). The four individuals derived from canine hosts demonstrated consistent *T. krabbei* affiliation based on the morphology of the rostellar hooks and the structure of the proglottids (6, 11). Parts of the same specimens were also stored in 96% alcohol for molecular analyses, using which we attempted to determine the cestode species based on the mitochondrial sequence data.

### DNA extraction, amplification and sequencing

A small part of *Taenia* spp. tissue (approximately 200 mg) was cut into small pieces and digested using the QIAamp DNA Mini Kit (Qiagen, Germany) according to the manufacturer's instructions.

The mitochondrial cytochrome c oxidase I (*cox1*) (approximately 463 bp) was amplified using the primers Cox1F: TGA-TCC-GTT-AGG-TGG-TGG-TGA, Cox2R: ACC-CTA-ACG-ACA-TAA-CAT-AAT-GAA-AATG, following the protocol adapted from Tull et al. (14). The total PCR volume of 25 μl contained 5 μl of parasite genomic DNA, 1X DreamTaq Green Buffer, 1 mM MgCl2, 200 μM of each dNTP, 500 nM of each primer, and 2.5 U DreamTaq DNA polymerase (Qiagen, Germany).

Each PCR consisted of 35 cycles of denaturation at 95 °C for 60 s, annealing at 50 °C for 60 s, and extension at 72 °C for 60 s; an initial denaturation phase consisting of incubation at 95 °C for 5 min and a final extension phase consisting of incubation at 72 °C for 5 min were also included. The PCR products were visualized on a 1.5% agarose gel stained with GoodView™ Nucleic Acid Gel Stain (SBS Genetech, Beijing, China). The amplicons were then excised from the gel, purified using the GenElute Gel Extraction Kit (Sigma, St. Louis, MO, USA), and subjected to bidirectional sequencing.

DNA sequencing was performed on an automated DNA sequencer, ABI Prism 3700, at the University of Veterinary Medicine and Pharmacy, Košice, Slovakia, on five cestode samples (two PCR replicates were amplified for each isolate). Obtained DNA sequences were compared with reference sequences in the GenBank database using the BLASTn nucleotide program. Sequences were clustered by similarity and a multiple alignment was generated for each cluster using the Clustal Omega algorithm (45), with the MView tool used to visualize the multiple alignments.

### Sequence alignments and phylogenetic analysis

Selected nucleotide substitution models were fitted using the maximum likelihood (ML) method and then ranked based on the Bayesian Information Criterion scores in MEGA v12.0.11. The Hasegawa–Kishino–Yano model with gamma-distributed rate variation and proportion of invariant sites (HKY+G+I) showed the best fit. Phylogenetic relationships were subsequently inferred using Bayesian inference (BI) and Neighbor-Joining (NJ) approaches to increase confidence in the resulting topology. *Echinococcus oligarthra* (15) was chosen as the outgroup for both analyses. The BI was performed using MrBayes v3.2.6 (16) and implemented in Geneious Prime v2025.1.2. Posterior probabilities were calculated using four Metropolis-coupled Markov chains (MCMC), each with 1,000,000 generations, and sampling every 200 generations. The first 25% of the samples were discarded as burn-in. The HKY+G+I nucleotide substitution model was implemented for the analysis. For the NJ analysis, the tree was constructed using the Geneious Tree Builder with the HKY genetic distance model and the neighbor-joining algorithm. Nodal support for the NJ topology was assessed through a bootstrap analysis with 100,000 pseudoreplicates. Finally, the resulting consensus tree topology was graphically formatted for publication using Inkscape v1.4.2.

Intraspecific (*p*_*intra*_) and interspecific (*p*_*inter*_) genetic distances were calculated using the p-distance method in MEGA v12.0.11 software. The calculations were performed on an alignment of 71 nucleotide sequences (5 sequences from Slovak carnivore tapeworms, 65 reference sequences retrieved from GenBank, 1 outgroup sequence), resulting in a final length of 396 nucleotides, which was the length adjusted to ensure accurate alignment and phylogram construction based on available GenBank resources. Standard error (SE) estimates of the mean distances were calculated using a bootstrap method with 500 replicates.

Nucleotide sequences of the examined Slovak samples from canids were deposited in GenBank under accession numbers PV857610-3; the nucleotide sequence of the Slovak felid sample was deposited in GenBank under accession number PV857714.

## Results

**Figure 1:**
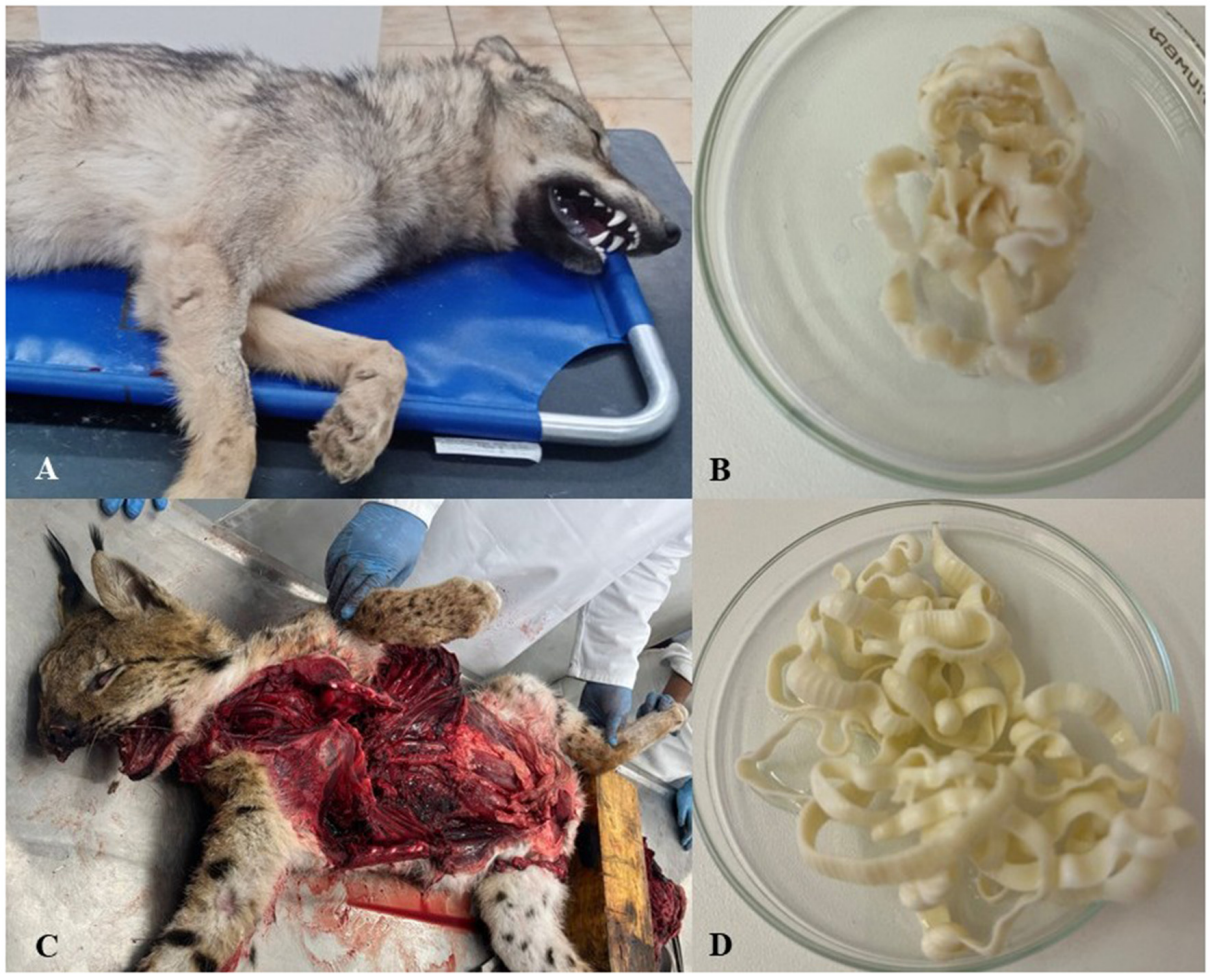
The carcasses of hosts and fidings of their taenias. **(A)**, The carcass of an adult gray wolf male (*Canis lupus* Linnaeus, 1758) from Bardejov district (near the village Beloveža). **(B)**, The tapeworm *Taenia krabbei* (44) found in wolf intestine. **(C)**, The carcass of an adult Eurasian lynx female (*Lynx lynx* Linnaues, 1758) from the Tatra national park (Poprad – Starý Smokovec). **(D)**, The tapeworm *Taenia lynciscapreoli* (11) found in lynx intestine.

All the carnivores examined were parasitized by taeniids. Two wolves were found to have 13 (in wolf from Bardejov district) and 21 adult specimens of cestodes (in individual from Poprad district), respectively; 15 in the jackal, 27 in the feral dog, and three in the lynx (Table 1; recovery from carcasses is documented in Figure 1). Five tapeworm samples (one selected from each positive host) obtained from canids and felids were examined by sequencing of the *cox1* gene (396 bp) and subsequent phylogenetic analysis.

**Table 1:** Recorded tapeworms – their hosts, intensity of infection and DNA sequences data.

*Taenia* species	Host species	Intensity of infection	DNA sequence bp obtained	Accession number deposited in GenBank
***Taenia krabbei***	Gray wolf (Bardejov district)	13	463 bp	PV857610
	Gray wolf (Poprad district)	21	463 bp	PV857611
	Feral street dog (Košice district)	27	463 bp	PV857612
	Golden jackal (Košice district)	15	463 bp	PV857613
***Taenia lynciscapreoli***	Eurasian lynx (Poprad district)	3	396 bp	PV857714

**Figure 2:**
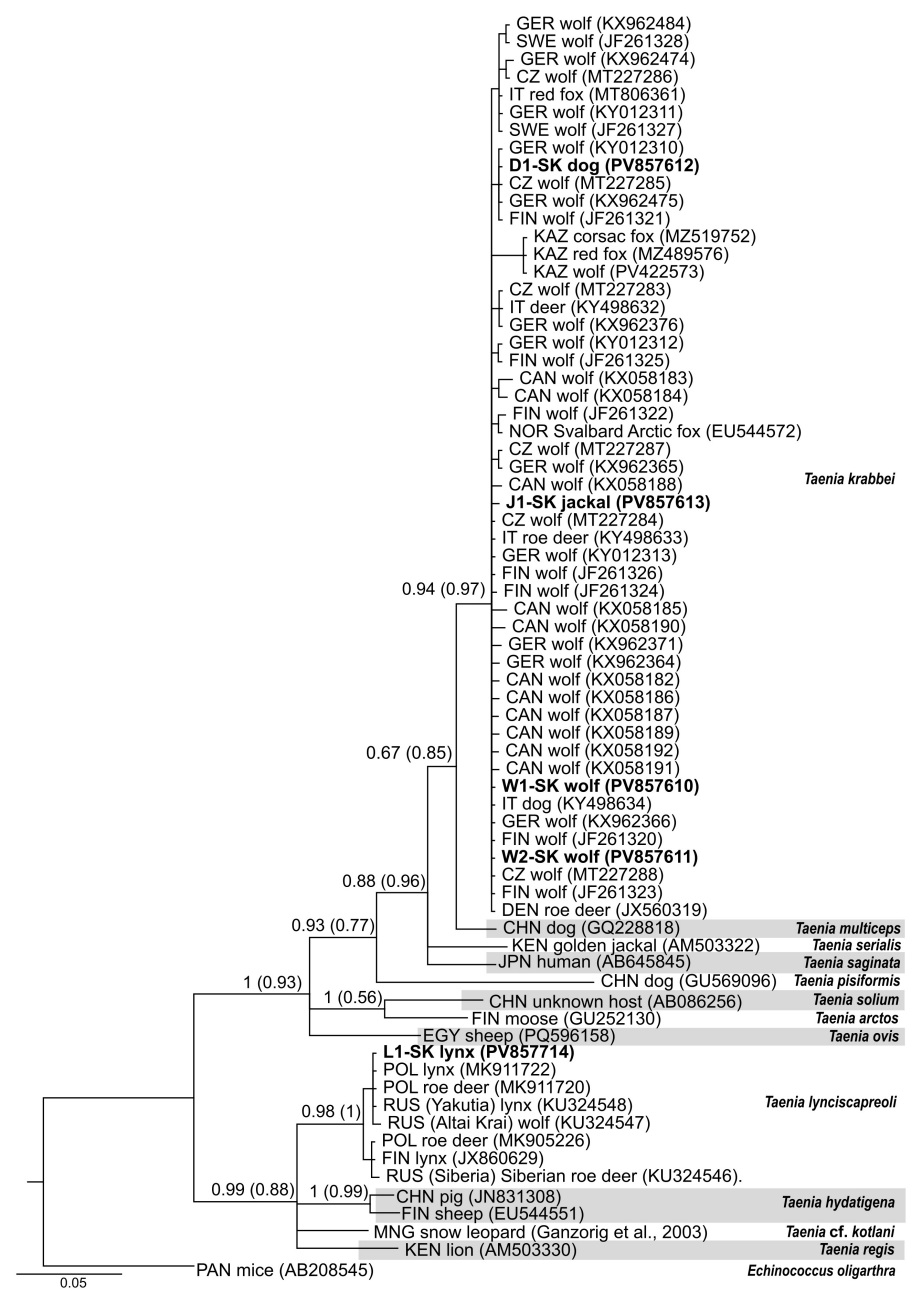
Phylogenetic tree of the genus *Taenia* inferred from partial mitochondrial *cox1* sequences (396 bp) of Slovak isolates of *Taenia krabbei* (44) and *Taenia lynciscapreoli* (11) recorded in the present study and selected references of *Taenia* spp. The obtained sequences (derived from Slovakia) are shown in boldface; reference sequences from GenBank are in regular font. For reference sequences of *T. krabbei* and *T. lynciscapreoli*, one representative sequence was chosen for each country/host combination to reflect genetic diversity. *Echinococcus oligarthra* (47) isolated from *Mus musculus* Linnaeus, 1758 from Panama (GenBank accession number AB208545) was determined as an outgroup. The isolate's countries of origin are indicated by abbreviations: CAN, Canada; CHN, China; CZ, Czech Republic; DEN, Denmark; EGY, Egypt; GER, Germany; FIN, Finland; IT, Italy; KAZ, Kazakhstan; KEN, Kenya; MNG, Mongolia; NOR, Norway; POL, Poland; RUS, Russia; SK, Slovakia; SWE, Sweden. The phylogram shown was generated using Bayesian inference. The scale bar indicates the estimated number of nucleotide substitutions per site. Nodal support values are presented as Bayesian posterior probabilities, followed by neighbor-joining bootstrap values in parentheses.

The isolate obtained from the Eurasian lynx (coded as L1-SK), found in the Tatra National Park in northeastern Slovakia, was identified as *T. lynciscapreoli*. As shown in phylogram (Figure 2), high posterior probability for BI and bootstrap values for NJ (0.98 and 1.00, respectively) were obtained for the group consisting of *T. lynciscapreoli*, which clearly distinguish the cluster from the most closely related species *T. hydatigena* Pallas, 1766, *Taenia regis* (46) and *Taenia* cf. *kotlani*, derived from canids and felids. The L1-SK isolate shared identical sequences with isolates originating from lynx and roe deer in Poland (GenBank accession numbers MK911722, MK911720), and with an isolate from a lynx in Yakutia, Russia (KU324547). The nucleotide sequences of haplotypes ascribed to *T. lynciscapreoli* differ only slightly (in 0.3–1.0% of nucleotides, intraspecific genetic distance *p*_*intra*_: 0.0040 ± 0.0021).). In contrast, the nucleotide divergences between *T. lynciscapreoli* and *T. hydatigena, T. regis, T*. cf. *kotlani* were considerably higher, ranging from 7.6 to 8.3% nucleotides (interspecific genetic distances *p*_*inter*_ between *T. lynciscapreoli* and other taxa were 0.074 ± 0.012 for *T. hydatigena*, 0.078 ± 0.014 for *T. regis*, and 0.071 ± 0.013 for *T. cf. kotlani*).

The L1-SK sequence was placed in a subclade with isolates from Poland collected from five Eurasian lynx (represented by the GenBank accession No. MK911722), three European roe deer (MK911720), and the two Russian (Siberian) isolate from the Eurasian lynx (KU324548) with identical sequences, and the Russian (Siberian) isolates from the gray wolf (KU324547) that had a single nucleotide substitution 27C/T.

The four isolates obtained from canids, specifically from the gray wolf (obtained from the districts Bardejov and Poprad in northeastern Slovakia), golden jackal and feral dog (obtained from the two sites in the district Košice) belonged to the *T. krabbei* (Figure 2). The Slovak samples under study grouped with other *T. krabbei* isolates into a monophyletic cluster, which had a posterior probability for BI of 0.94 and a bootstrap value for NJ of 0.97. The percentage of sequence identity between the isolates of *T. krabbei* was high (99.2%−100%, *p*_*intra*_: 0.0083 ± 0.0017), while the identity with the genetically closest species *T. multiceps* (Leske, 1780), an intestinal parasite of canine carnivores, was markedly lower (95.4–95.9%, *p*_*inter*_: 0.045±0.010).

Two adult isolates obtained from wolves (designated as W1-SK and W2-SK), were identical in *cox1* sequences, except for position 45, where base G was present in W1-SK, while in W2-SK the two overlapping peaks (A, G) with almost equal intensity were detected in forward and reverse screening. This could likely be attributed to heteroplasmy - the presence of more than one mitochondrial type in the same individual (17). The ambiguous nucleotide symbol R (purine base, which can be either adenine - A, or guanine - G), labeled according to IUPAC nomenclature, was therefore used at this site for sequence evaluation. The wolf isolates shared identical nucleotide sequences with isolates from European definitive hosts - wolves from Finland and Germany (JF261320, KX962366) and a dog from Italy (KY498634), originated from the area of the Northern Apennines, where the wolf population was recently re-established (7).

The investigated jackal isolate SK-J1 was most similar to wolf isolates derived from the Czech Republic and Germany (MT227284, KY012313) and roe deer in Italy (KX498633), from which it differed by a single nucleotide substitution 49 A/G. The investigated dog isolate D1-SK was placed in a subclade together with wolf isolates from Germany (KX962475, KY012310), the Czech Republic (MT227285) and Finland (JF261321).

## Discussion

This study recorded the first occurrence of the tapeworm *T. lynciscapreoli* in Slovakia, which was found in the Eurasian lynx, a feline carnivore, in the Tatra National Park. In addition, four isolates taken from canine hosts (gray wolf, golden jackal and domestic dog) in eastern Slovakia showed a genetic structure corresponding to *T. krabbei*.

After the primary indication of the species affiliation using morphological characters, molecular methods demonstrated an unambiguous classification to the above mentioned cestode species. According to the rule of the DNA barcoding gap, interspecific genetic distances significantly exceed intraspecific genetic distances for the selected barcode region (18). On this account, Zhang et al. (19) indicated that DNA barcoding using short fragments of *cox1* can distinguish taeniid species at an optimal species threshold of 2.0%, which conforms to our two species (*T. lynciscapreoli, T. krabbei*) recorded in the present study.

The genetically confirmed record of *T. lynciscapreoli* found in the intestines of the Eurasian lynx in Tatra National Park in the present study represents the first finding of the species in Slovakia. The species *T. lynciscapreoli* was first taxonomically described relatively recently, in 2016 (11). Its validation was preceded by differentiation of *T. lynciscapreoli* from other taeniids in Holartic using mitochondrial markers and morphology of rostellar hooks in isolates from the European lynx by Lavikainen et al. (20). *Taenia lynciscapreoli* adult specimens have been recorded from the intestines of the Eurasian lynx and wolf in Finland, Poland and Russia, and within intermediate hosts, metacestodes were reported in the lungs of the European roe deer, Siberian roe deer, semi-domesticated reindeer, and moose/Eurasian elk in Finland, Poland, Russia and Sweden (4, 8, 11, 21–23).

Previous molecular studies together with the presented phylogenetic analysis indicated that *T. lynciscapreoli* is closely related to *T. hydatigena, T. regis* and *T*. cf. *kotlani* (11, 20, 23–25). Similarities between these taeniids in life cycle, with intermediate hosts being wild and domestic ungulates (cervids and bovids) and definitive hosts being felids and canids, together with similarity in mitochondrial DNA sequences suggest that they may have evolved from a common ancestor and that in the case of *T. hydatigena*, a host-switching from felids to canids may have occurred during the evolutionary history (1).

The occurrence of *T. lynciscapreoli* depends on the presence of its main definitive and intermediate hosts, lynx and roe deer, which have an almost transcontinental and overlapping distribution in Eurasia (11). As a small cervid, roe deer are the preferred prey for lynx, thus maintaining the transmission of the life cycle, and occasional infections of larger cervids (from the genera *Alces, Cervus*) with the *T. lynciscapreoli* metacestodes were recorded mostly in areas where this cycle occurs (11, 26). For Slovakia, it has been estimated that ungulates represent 89.8% of the total weight of food consumed by lynx, of which roe deer (66.9%) is the main prey (27, 28).

The autochthonous population of Carpathian lynx inhabits mainly mountainous regions of Slovakia, where it prefers vertically rugged terrain with plenty of shelter and rocky slopes (29). The current size of the lynx population in the country, adjusted including subadults and cubs (which constitute approximately 35% of the population), is estimated at approximately 400 individuals, and appears to have remained relatively constant over the past two decades (30). The number of lynxes listed in the Hunting Statistical Yearbook of the Slovak Republic (HSYSR) 2024 (31) in Slovak hunting grounds in spring were estimated at 1,837 individuals (including multiple records due to the migration of felines). The 517 lynxes observed in the Prešov region (which includes the Tatra National Park, *T. lynciscapreoli* sampling site) in 2024 represent 28.1% of the national census, which is significantly higher than the ratio of the region's area to the entire territory of Slovakia (18.3%). The reported roe deer population (14,932 individuals) in the Prešov region constituted a similar share (14.3%) of the total deer population as was the size of the area relative to the territory of the whole country. This region thus seemingly provides suitable conditions for sustaining the transmission cycle of *T. lynciscapreoli* between the abundant lynx and roe deer (preferred intermediate host). In the area of the Tatra National Park, where the lynx was found, its current number is estimated at 10–20 individuals, and it is most commonly found in the foothills and mountain regions, where its main prey, the roe deer, also lives (30).

In addition, based on *cox1* data, adult *T. krabbei* tapeworms were diagnosed in two gray wolves, one golden jackal, and one feral dog in southeastern (Košice district) and northeastern Slovakia (Bardejov and Poprad districts). These findings represent the first records of *T. krabbei* in jackal and dog in Slovakia. To our knowledge, *T. krabbei* was not documented before also in the European jackal population; the only record found in the literature was from Azerbaijan, South Caucasus region, where the species was recorded in one out of 114 jackals (i.e., with a prevalence of 0.8) in the early 1980s (32).

In continental Europe, *T. krabbei* primarily uses wolves as definitive hosts, and is rarely found in other canids (6, 33). The metacestodes are usually seen in the cardiac and skeletal muscles of intermediate hosts, among which cervids serve as principle intermediate hosts (34, 35). Although *T. krabbei* is not considered a public health concern (36), the formation of numerous cysts after extensive infection can make game meat visually unattractive due to aesthetic reasons and is often leads to its discard (7). In Slovakia, between 2002 and 2007, adult *T. krabbei* were found in one wolf (out of 6 wolves examined, prevalence *P* = 16.6%), three red foxes (out of 302 foxes, *P* = 0.9%), and cysticerci were observed in the myocardium of one red deer (out of 61 deer, *P* = 1.6%) (10).

The four Slovak isolates examined in this study differed by one to three nucleotides in the 396 bp region of *cox1* and were distributed in the phylogram at different positions of the cluster attributed to *T. krabbei*, according to their definitive hosts (wolf, dog, jackal). The sequences of this taeniid species, which have been preserved in the Slovak region thanks to the Carpathian wolf population (37), showed the highest affinity with isolates from European wolves from the Czech Republic, Germany (hosts belonging to the Central European lowland population), Finland (Scandinavian/Karelian population), and Italy (Italian peninsula population) (8, 9, 38).

Although the wolf has been recognized as the original definitive host of *T. krabbei*, the role of alternative definitive hosts, such as domestic dogs, red foxes, raccoon dogs, and golden jackals, should not be underestimated and may contribute to the dispersal and maintenance of the infection in the given area (38, 39). In this study, half of the *T. krabbei* samples were collected from definitive hosts other than wolves. The presented finding of the dog-derived isolate (Genbank accession number PV857612) indicated the epidemiological importance of this host in the life cycle of *T. krabbei*. Hunting dogs, sheepdogs, and stray dogs in particular can play an important role in the spread of cysticercosis to domestic and/or wild ruminants (7, 34, 40).

Since the early 1980s, the golden jackal has spread rapidly throughout Europe, with Balkan populations spreading mainly to Central Europe, and its range continues to expand (41). In Slovakia, spring populations of jackals have been reported since 2009. While in 2009 only eight individuals were counted in hunting grounds and three individuals were shot, in 2024, as many as 1,063 individuals were reported and 130 animals were shot, which corresponds to a 133-fold and 43-fold increase during this period (31). Interestingly, approximately two-thirds (67.2%, 714/1063) of the observed jackals came from hunting grounds in the Košice region (southeastern Slovakia), where the jackal-derived isolate (GenBank accession number PV857613) was found, with the highest population density along the Bodrog, Hornád, Latorica, Ondava, and other rivers (42, 43).

## Conclusions

This study documented the presence of the tapeworm *T. krabbei* in gray wolves, golden jackal, and domestic dog in eastern Slovakia. Wolves are considered the primary definitive hosts of this tapeworm, but our findings also indicate the epidemiological importance of non-wolf hosts in maintaining its life cycle. Further, the presence of the recently described species *T. lynciscapreoli* in European lynxes has been confirmed in Slovakia, adding to knowledge of its occurrence in central Europe, and following the recognized distribution in some Scandinavian countries and Russia in the European context. These findings suggest the need to control these parasitic diseases, especially in hunting, herding, and stray dogs, which can play an important role in spreading cysticercosis to domestic and game animals.

## Data Availability

The datasets presented in this study can be found in online repositories. The names of the repository/repositories and accession number(s) can be found below: https://www.ncbi.nlm.nih.gov/genbank/, PV857610, PV857611, PV857612, PV857613, PV857714.
